# Renin-Angiotensin-Aldosterone System Inhibitors in COVID-19: A Review

**DOI:** 10.6061/clinics/2021/e2342

**Published:** 2021-03-30

**Authors:** Filipe Ferrari, Vítor Magnus Martins, Flávio Danni Fuchs, Ricardo Stein

**Affiliations:** I Programa de Pos-Graduacao em Cardiologia e Ciencias Cardiovasculares, Hospital de Clinicas de Porto Alegre, Universidade Federal do Rio Grande do Sul, Porto Alegre, RS, BR; II Hospital de Clinicas de Porto Alegre, Porto Alegre, RS, BR; III Divisao de Cardiologia, Hospital de Clinicas de Porto Alegre, Universidade Federal do Rio Grande do Sul, Porto Alegre, RS, BR; IV Faculdade de Medicina, Universidade Federal do Rio Grande do Sul, Porto Alegre, RS, BR

**Keywords:** Pandemic, Infection, SARS-CoV-2, Hypertension, Heart Failure

## Abstract

Among the multiple uncertainties surrounding the novel coronavirus disease (COVID-19) pandemic, a research letter published in The Lancet implicated drugs that antagonize the renin-angiotensin-aldosterone system (RAAS) in an unfavorable prognosis of COVID-19. This report prompted investigations to identify mechanisms by which blocking angiotensin-converting enzyme 2 (ACE2) could lead to serious consequences in severe acute respiratory syndrome coronavirus 2 (SARS-CoV-2). The possible association between RAAS inhibitors use and unfavorable prognosis in this disease may have been biased by the presence of underlying cardiovascular diseases. As the number of COVID-19 cases has increased worldwide, it has now become possible to investigate the association between RAAS inhibitors and unfavorable prognosis in larger cohorts. Observational studies and one randomized clinical trial failed to identify any consistent association between the use of these drugs and unfavorable prognosis in COVID-19. In view of the accumulated clinical evidence, several scientific societies recommend that treatment with RAAS inhibitors should not be discontinued in patients diagnosed with COVID-19 (unless contraindicated). This recommendation should be followed by clinicians and patients.

## INTRODUCTION

In March 2020, a research letter published in The Lancet suggested that patients with hypertension, heart disease or diabetes who were on renin-angiotensin-aldosterone system (RAAS) inhibitors, such as angiotensin-converting enzyme (ACE) inhibitors or angiotensin II receptor blockers (ARBs), might be at greater risk of severe disease with severe acute respiratory syndrome coronavirus 2 (SARS-CoV-2) infection (1). This hypothesis was based on the fact that SARS-CoV-2, the causative agent of COVID-19, must bind to ACE2 for entry into cells. Since RAAS inhibitors can increase ACE2 levels, their use could facilitate viral entry, and thus lead to a worse prognosis.

Several observational studies in patients with COVID-19 conducted worldwide, including in China (2-6), Italy (7-11), the United States (12-14), South Korea (15,16), Korea (17-19), Saudi Arabia (20), Sweden (21), Spain (22), London (23), Scotland (24), Turkey (25,26), and Denmark (27) did not demonstrate any association between the use of these drugs and unfavorable outcomes. These data were corroborated by a randomized clinical trial (RCT) conducted in Brazil (28). RAAS inhibitors are cornerstones in the treatment of several common cardiovascular diseases, including hypertension (29), heart failure (30), and myocardial infarction (31); thus, caution is warranted before considering their discontinuation.

In this review, we discuss the relationship between the use of ACE inhibitors or ARBs and the clinical course of SARS-CoV-2 infection and address the importance of careful assessment of the risks and potential benefits of using these agents in the context of the COVID-19 pandemic.

## METHODS

We searched literature published in PubMed/MEDLINE from January 1, 2020 to November 26, 2020, regarding the use of RAAS inhibitors and outcomes in patients with COVID-19. We excluded studies from the MedRxiv database, since manuscripts from this source are not peer-reviewed. The following research terms were utilized: (“*coronavirus*” OR “*COVID-19*” OR “*Severe acute respiratory syndrome coronavirus 2*” OR “*2019-nCoV*” OR “*SARS-CoV-2*” OR “*SARS-CoV*“) AND (“*Renin-angiotensin-aldosterone system inhibitors*” OR “*RAAS inhibitors*” OR “*ACE inhibitors*” OR “*Angiotensin II receptor blockers*“). Ongoing RCTs registered at ClinicalTrials.gov were identified. We manually searched the reference lists of all included studies to identify other potential articles. There were no language restrictions.

The initial search identified 287 titles and abstracts, of which five were excluded as duplicates. Thus, we evaluated 282 titles and abstracts, of which 233 were excluded (six published in the MedRxiv database). Therefore, 50 potentially eligible studies were read in full, of which 14 were excluded. The types of reports excluded were as follows: studies that compared ACE inhibitors *vs.* ARBs (N=1), review articles (N=6), letters for editor (N=4), comments (N=2), and others (N=1). Four eligible studies (2,3,11,27) were identified in the reference lists of other studies and one on a cardiology portal (28). In total, 40 articles were included in the review (2-28,32-44).

### Hypertension, Cardiovascular Disease, and COVID-19

Current evidence indicates that older patients with underlying chronic diseases (*e.g.*, hypertension and cardiovascular diseases) who are affected by COVID-19 constitute a group with higher mortality risk (45-47), which may be responsible for a greater susceptibility of these patients to myocardial involvement in SARS-CoV-2 infection (48). In addition, patients with established cardiovascular disease may be more prone to severe or fatal SARS-CoV-2 infection (47). In a large series of patients hospitalized with COVID-19, the proportion of patients with hypertension and chronic heart disease ranged from 5% to 64% and 3% to 43%, respectively (46-82). Clinical characteristics of patients with COVID-19 are presented in Table 1.

A study on 1,099 patients with COVID-19 showed that 24% had at least one comorbidity (*e.g.*, hypertension) (47). In contrast, in a Chinese study involving more than 2,200 individuals with COVID-19, patients with hypertension represented 20% of the sample (N=440), while those with established cardiovascular disease accounted for approximately 7% (N=154) (75). Wang et al. (46) reported that, among 138 hospitalized patients with COVID-19 in China, more than 30% had hypertension. When patients who needed intensive care unit (ICU) support were evaluated, almost 60% had hypertension, compared with 22% of those who did not require ICU care (46). In addition, in a case series of adults with COVID-19, the proportion of individuals with hypertension who did not need mechanical ventilation was lower than those who did (70). Similar data have been observed in patients with coronary artery disease (70). Sun et al. (62) analyzed 244 Chinese individuals with COVID-19 and found that the proportion of patients with hypertension was higher among those who died than among those who recovered (63.3% *vs.* 50.4%, respectively, *p*=0.042). An analysis of 162 hospitalized patients in Israel showed that, as disease severity increased, so did the proportions of patients with hypertension (mild, 19.6%; moderate, 40.9%; severe, 50%) and ischemic heart disease (mild, 5.4%; moderate, 6.8%; severe, 15.4%) (77).

A meta-analysis of seven studies, including 1,576 patients with COVID-19, indicated that those with the most severe disease had a higher risk of having hypertension, with an odds ratio (OR) of 2.36 [95% confidence interval (CI), 1.46 to 3.83), and cardiovascular disease, with an OR of 3.42 (95% CI, 1.88 to 6.22) (63). Wu et al. (66) also found a higher risk of death and acute respiratory distress syndrome in hypertensive patients with COVID-19. In another meta-analysis, hypertension was associated with up to 2.5-fold higher risk of having more severe disease (OR, 2.49; 95% CI, 1.98 to 3,12), as well as a higher mortality risk (OR, 2.42; 95% CI, 1.51 to 3.90) (84).

### SARS-CoV-2 and the Renin-Angiotensin-Aldosterone System

**Figure 1:**
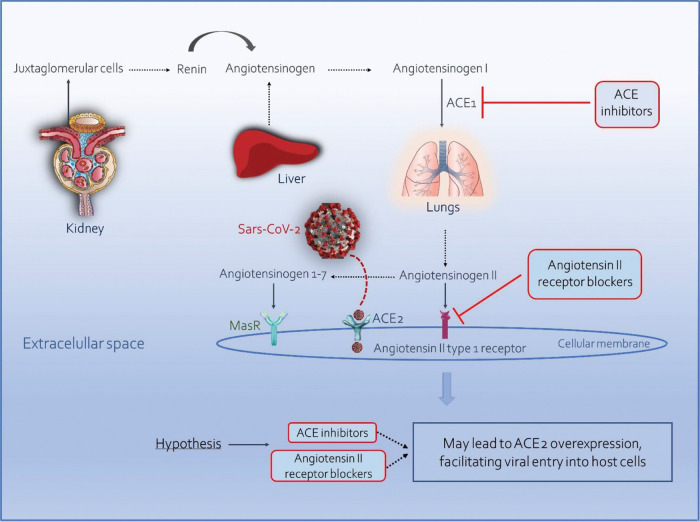
Renin-angiotensin-aldosterone system and drugs that act on this system. MasR: Mas receptor; ACE1: ACE2: Angiotensin-converting enzyme 2.

RAAS exerts key physiological functions in the homeostasis of the cardiovascular and renal systems (85). This complex pathway begins with the release of renin by the juxtaglomerular cells, catalyzing the conversion of angiotensinogen into angiotensin I. This is subsequently converted into angiotensin II in the lungs and kidneys by ACE. Angiotensin II, in turn, is transformed into angiotensin 1-7 by ACE2 (86) (Figure 1).

The finding that the use of ARBs can increase the expression of ACE2 led to the hypothesis that patients on such therapy might be more susceptible to infection with SARS-CoV-2, which has an affinity for this enzyme (87) (Figure 1). For instance, Soro-Paavonen et al. (88) showed that patients with diabetes who were on ACE inhibitors had increased circulating levels of ACE2.

However, a competing hypothesis suggests a beneficial effect of ACE inhibitors or ARBs in patients with COVID-19. According to this hypothesis, the use of these drugs could decrease the production of angiotensin II and increase the generation of angiotensin 1-7 through ACE2 and activation of the Mas receptor, which might play a role in reducing inflammation and pulmonary fibrosis (89,90). Angiotensin II, in turn, can lead to lysosomal internalization of ACE2, causing the expression of ACE2 to be reduced; the use of losartan can prevent this effect through its action on ACE2 via AT1 receptors. Thus, ARBs may reduce SARS-CoV-2 entry into cells. However, a virion needs only one receptor to infect a cell, and the effect of ARBs on the breakdown of angiotensin II to angiotensin 1-7 is still unknown (91).

The relationship between SARS-CoV-2, RAAS inhibitors, ACE2, and a higher risk of infection remains controversial. Current data are very limited and do not provide certainty to support or refute the aforementioned assumptions and concerns. The inferior prognosis of COVID-19 observed in patients with chronic diseases (such as hypertension) may have been simply due to the comorbidities themselves, not due to therapy with RAAS inhibitors. Considering that the prevalence of hypertension increases considerably with advancing age (92), and that the elderly population is at a particularly high risk of complications from COVID-19 (93), associations between hypertension, RAAS inhibitors, and inferior prognosis of COVID-19 may not necessarily be causal.

### COVID-19 and Renin-Angiotensin-Aldosterone System Inhibitors

#### Evidence of Neutral Effect Based on Observational Studies and in a Randomized Clinical Trial

A population-based case-control study (10) with data from 6,300 patients and 31,000 controls from the Lombardy region in Italy, found no association between the use of ACE inhibitors or ARBs and SARS-CoV-2 infection among overall COVID-19 patients or patients with severe or fatal disease. Reynolds et al. (14) evaluated 12,594 individuals who were tested for COVID-19, of whom 4,357 had hypertension. They further analyzed the relationship between treatment with five classes of antihypertensive drugs, including ACE inhibitors (22% of patients) and ARBs (28% of patients), and the probability of a positive or negative COVID-19 test result. No association was observed between the use of RAAS inhibitors and the risk of a positive test result for COVID-19 (14). A study conducted in Saudi Arabia found no differences in ICU admission, ICU admission within 24 hours of hospitalization, ICU stay (days), and ICU death (*p*=0.19; *p*=0.23; *p*=0.13; *p*=0.58, respectively) (20) among patients receiving ACE inhibitors/ARBs *vs.* non-ACE inhibitors/ARBs.

A retrospective cohort study conducted in the state of Florida, U.S assessed whether there was an association between ACE inhibitors or ARBs and the likelihood of SARS-CoV-2 infection (40). Approximately 19,000 individuals were tested for COVID-19; among them, 12% used ACE inhibitors or ARBs. Among these patients, 421 tested positive and were admitted to the hospital, 161 were admitted to the ICU, and 111 required mechanical ventilation (40). In Belgium, De Spiegeleer et al. (36) also found no differences between therapy with ACE inhibitors/ARBs and non-ACE inhibitors/ARBs in asymptomatic patients or those with severe clinical outcomes.

A retrospective case series analyzed data from approximately 1,200 patients hospitalized with COVID-19 in China (2). Among individuals with severe and non-severe disease, the proportions of hypertensive patients using ACE inhibitors and ARBs did not differ. The same was observed between non-survivors and survivors, suggesting that these drugs are not associated with severity of disease or mortality in COVID-19 among hypertensive patients. A Danish retrospective study evaluated approximately 4,500 patients with COVID-19, 895 of whom received ACE inhibitors or ARBs. No association with mortality or severe illness was observed among patients receiving these medications (27). In Italy, Bravi et al. (33) conducted a retrospective case-control study on adults with COVID-19 (N=1,603), who were followed for a median of 24 days. Multivariate analysis revealed no association between the use of ACE inhibitors or ARBs and disease severity among 543 hypertensive patients using these drugs. Male sex, age, and diabetes were the lone predictors of more severe disease. Data from China corroborate these findings. Patients on ACE inhibitor or ARB therapy (N=40) or other classes of antihypertensive drugs (N=61) were compared. No differences were observed between groups in terms of hospital mortality, requirement of ICU support, or invasive mechanical ventilation (42).

Sardu et al. (11) analyzed the responses of ACE inhibitors *vs.* ARBs *vs.* calcium channel blockers in 62 hypertensive patients with COVID-19, and found no association of the use of these drugs with requirement of mechanical ventilation, requirement of ICU support, cardiac injury, and mortality. Finally, in another study comparing ACE inhibitors/ARBs *vs.* calcium channel blockers in COVID-19, no differences were observed in the chest CT improved time and hospital stay between groups (3).

The most important evidence in this scenario is based on the first and only RCT to date: The continuing *versus* suspending ACE inhibitors and angiotensin receptor blockers: Impact on adverse outcomes in hospitalized patients with severe acute respiratory syndrome coronavirus 2 (SARS-CoV-2) (BRACE CORONA) trial (28). This study, conducted in Brazil, randomized 659 patients hospitalized with COVID-19 (mean age: 55 years) for temporary suspension of ACE inhibitor/ARB therapy (N=334) or continuation of ACE inhibitor/ARB therapy (N=325). The primary outcome was the number of days patients were alive and out of the hospital during a follow-up of 30 days, and the secondary outcome was the number of all-cause deaths at 30 days. There were no differences in the primary and secondary outcomes between groups (21.9% *vs.* 22.9%; *p*=0.09; 2.7% *vs.* 2.8%; *p*=0.95, respectively). This study confirmed the previous data presented in several observational studies, showing that there is no clinical benefit of interruption of these drugs in patients hospitalized with COVID-19.

A detailed summary of aforementioned studies is presented in Table 2.

### Evidence of Potential Benefits Based on Observational Studies

In Madrid, Spain, de Abajo et al. (35) conducted a population-based case-control study on patients diagnosed with COVID-19. When users of other antihypertensive drugs were compared to users of RAAS inhibitors, no increased risk was observed with ACE inhibitors or ARBs. Interestingly, they observed a reduction in the odds of hospital admission in diabetic patients receiving RAAS inhibitors. Meng et al. (94) studied hypertensive patients with COVID-19, who were divided into a RAAS inhibitor group (ACE inhibitors/ARBs, N=17) and other antihypertensive agent group, including calcium channel blockers, beta-blockers, and diuretics (N=25). Patients using ACE inhibitors or ARBs showed an increase in CD3^+^ and CD8^+^ T-cell counts, in addition to a lower frequency of severe illness and a trend towards lower interleukin-6 levels in peripheral blood. These findings point to new pathways that may explain the possible benefits of the use of these drugs in hypertensives with COVID-19.

Felice et al. (37) conducted a study on 133 hypertensive patients diagnosed with COVID-19 at a single center in Italy. They were divided into one group of those taking ACE inhibitors (N=40; 70% taking ramipril) or ARBs (N=42, more than 50% taking olmesartan) and another group of patients using non-RAAS inhibitors (N=51). Patients on long term therapy with RAAS inhibitors had lower odds of admission to semi-intensive or intensive care than those treated with non-RAAS inhibitors. Another observational study conducted in Italy (Rozzano-Milan, Lombardy) suggested a possible mortality reduction in patients using ACE inhibitors/ARBs (34). In this study, approximately 400 patients with COVID-19 were divided into three groups: 1) patients who received continued ACE inhibitor/ARB therapy (14.1%), 2) patients who were discontinuation from ACE inhibitor/ARB therapy at hospitalization (due to hypotension, worsening of renal function, or other factors) (29.5%), and 3) patients who were not on RAAS inhibitors at baseline (56.4%). The primary outcome was mortality within 20 days of hospital admission. The mortality rates in these groups were 12.5%, 27.4%, and 17.4%, respectively (*p*=0.036), suggesting a reduction in this serious outcome in patients who continued the use of ACE inhibitors or ARBs, compared to those who discontinued the therapy (34). These findings were corroborated by another study, in which patients with hypertension were divided into RAAS inhibitor users (N=41) and non-users (N=241). Mortality was significantly lower in RAAS inhibitor users (41), suggesting a better prognosis of hypertensive patients on these medications.

Gao et al. (38) evaluated 2,900 hospitalized patients in China, of whom approximately 30% had hypertension. Hypertensive patients had a two-fold increase in the relative risk of mortality compared to normotensive patients. It is important to point out that those with a history of hypertension but not on antihypertensive treatment (N=140) had a significantly higher risk of mortality than those on antihypertensive treatment (N=730).

The association between in-hospital use of ACE inhibitors/ARBs and death over a 28-day period in patients with COVID-19 compared with non-ACE inhibitors/ARBs using propensity score matching was analyzed in another study (44). Nine hundred and six patients treated with an ACE inhibitor or ARB were matched with 1,812 individuals treated with non-ACE inhibitors/ARBs agents during hospitalization. In comparison to patients receiving non-ACE inhibitors/ARBs those receiving ACE inhibitors/ARBs demonstrated a lower risk of 28-day mortality due to COVID-19, as well as reduced all-cause mortality among patients with hypertension (adjusted HR 0.32; 95% CI, 0.15 to 0.66), hypertension combined with coronary artery disease (adjusted HR 0.11; 95% CI, 0.04 to 0.31), and coronary artery disease (adjusted HR 0.38; 95% CI, 0.16 to 0.89) (44).

Another study in Turkey identified 249 hypertensive patients with COVID-19. The risk of severe disease tended to be lower with the use of ARBs. Additionally, ACE inhibitor therapy showed a reduced risk of severe disease and lower hospitalization rates (26).

In a population cohort study (N=8.3 million), approximately 20,000 individuals had COVID-19, of which 1,286 required ICU support. After adjustment for demographic factors, comorbidities, and use of other drugs, ACE inhibitor/ARB utilization was associated with reduced risk of infection with SARS-CoV-2; association with increase in ICU admission was also not observed (39). In another large database analysis (N=1.4 million) conducted in Sweden, the risk of hospitalization/death due to COVID-19 in the general population was lower in patients receiving ACE inhibitors/ARBs, after adjustment for 45 variables (OR 0.85; 95% CI, 0.81 to 0.89). Specifically, in patients with COVID-19, a reduction in all-cause death was found in those receiving ACE inhibitors/ARBs (21).

A detailed summary of aforementioned studies is presented in Table 2.

Furthermore, Bean et al. (32) studied a cohort of 1,200 patients with COVID-19 in the United Kingdom. Of these, 33% were on ACE inhibitors or ARBs. These patients were older and had higher frequency of comorbidities than patients who did not take these medications. Hypertension, diabetes, and heart failure were present in 85%, 54%, and 16% *vs.* 38%, 25%, and 5% of patients in the two groups, respectively. The primary outcome of the study was defined as death or need for ICU admission within 21 days of symptom onset. The likelihood of the primary outcome was similar between groups (OR, 0.83; 95% CI, 0.64 to 1.07). However, after adjusting for age and sex, the probability of severe illness was significantly lower in patients on ACE inhibitors or ARBs (OR 0.70; 95% CI, 0.53 to 0.91; *p*<0.01). A higher frequency of treatment with statins in patients using ACE inhibitors/ARBs *vs.* patients not using ACE inhibitors/ARBs (67.2% *vs.* 25%) (32) may have been one of the factors influencing better outcomes in the former group. The use of statins was associated with better survival among patients with COVID-19 in a retrospective study which used data from 169 hospitals in three continents (95). However, The New England Journal of Medicine has retracted the article at the request of the authors (96), based on the consideration that none of the authors had access to the underlying data. This retraction occurred shortly after the medical journal raised concerns about the Surgisphere database underlying the study (97). In the rush to publish during the COVID-19 pandemic, a shortened time from submission to publication with a subsequent increase in preprints, before studies have been adequately peer-reviewed, has raised concerns about the integrity of information in recent research.

### What do the Meta-Analyses Indicate?

Meta-analyses of studies exploring the association between the use of RAAS inhibitors and unfavorable prognosis in patients with COVID-19 have been published (78-84). They were not all inclusive, but showed trends similar to those identified in the individual studies.

Flacco et al. (98) conducted a meta-analysis on ten studies comprising approximately 10,000 individuals with hypertension. When patients treated with ACE inhibitors or ARBs were compared to those who were not on these medications, there were no differences in the risk of severe or fatal COVID-19, either in relation to ACE inhibitors (OR 0.90; 95% CI, 0.65 to 1.26; *p*=0.55), or ARBs (OR 0.92, 95% CI, 0.75 to 1.12; *p*=0.41). In another meta-analysis, the users of ACE inhibitors/ARBs did not show increased risk of developing severe disease when compared to the non-users (OR 0.81; 95% CI, 0.41 to 1.58; *p*=0.53). In addition, while evaluating the risk of mortality, no association was observed with the use of ACE inhibitors/ARBs (OR 0.86; 95% CI, 0.53 to 1.41; *p*=0.55) (99). Similar findings were observed by Pranata et al. (100), where ACE inhibitors/ARBs use was not associated with any mortality increment (OR 0.73; 0.38 to 1.40) or disease severity (OR 1.03; 0.73 to 1.45).

The meta-analysis conducted by Pirola and Sookoian (101) included 16 studies on patients with COVID-19 (N=24,676) and compared patients with critical (n=4134) *vs.* non-critical (n=20,542) outcomes. A 24% reduction in the risk of death and/or critical illness was observed with the use of ACE inhibitors/ARBs (OR 0.76; 95% CI, 0.651 to 0.907) when compared to the non-ACE inhibitor/ARB group. A meta-analysis was conducted on nine observational studies on hypertensive patients (N=3936), all considered of high methodological quality. When comparing treatment with ACE inhibitors/ARBs *vs.* non-ACE inhibitors/ARBs, there was no association with disease severity (OR, 0.71; 95% CI, 0.46 to 1.08). In contrast, a lower mortality risk was observed with the use of ACE inhibitors/ARBs (OR 0.57; 95% CI, 0.38 to 0.84; *p*=0.004) (102). Finally, two other meta-analyses (103,104) found reduced mortality and reduced risk of severe COVID-19 in patients using ACE inhibitors/ARB medications.

### Recommendations from Scientific Societies

Several scientific societies concur in their risk assessment of RAAS inhibitors and have recommended that treatment with these drugs should be continued as usual during the COVID-19 pandemic. The Heart Failure Society of America, American College of Cardiology, American Heart Association (105), European Hypertension Society (106), Canadian Cardiovascular Society, Canadian Heart Failure Society (107), and the Heart Failure Department of the Brazilian Society of Cardiology (108) have all published position statements or official guidance emphasizing the importance of continuing therapy with these drugs, given their well-established clinical benefits and absence of reliable evidence of an association with severe COVID-19.

### Randomized Clinical Trials in Progress

The ACE inhibitors stopping in COVID-19 (ACEI-COVID-19) study is a multicenter RCT being conducted in Austria and Germany that is recruiting symptomatic patients with COVID-19 (estimated N=208) to evaluate the outcomes of interruption of ACE inhibitor or ARB treatment *vs.* continued use of these medications (NCT04353596).

In France, the ACE inhibitors or ARBs discontinuation in context of SARS-CoV-2 pandemic (ACORES-2) trial is expected to randomize more than 500 patients who will be divided into two groups: in one patients will continue using ACE inhibitors/ARBs and another in which treatment with these drugs will be discontinued. Finally, clinical risk reduction will be evaluated (NCT04329195).

Another ongoing RCT, conducted in Brazil, is also recruiting patients with COVID-19 to evaluate the impact of discontinuation of ACE inhibitors/ARBs on length of hospital stay and mortality. It is estimated that 500 patients will be randomized (NCT04364893).

These studies may provide definitive evidence of whether treatment with RAAS inhibitors predisposes patients with COVID-19 to an unfavorable prognosis.

## CONCLUSIONS

Despite the suggestion that the use of RAAS inhibitors could have deleterious biological consequences, these risks were not confirmed in several cohorts of patients with COVID-19, and by a Brazilian RCT; indeed, some observational studies have suggested that the use of these drugs could have beneficial effects. Other RCTs are in progress and may further clarify the effect of RAAS inhibitors on disease course and prognosis in COVID-19. Until further clarification, treatment with RAAS inhibitors should be continued as required in patients with SARS-CoV-2 infection and concomitant hypertension and/or cardiovascular disease.

## AUTHOR CONTRIBUTIONS

Ferrari F, Martins VM, Fuchs FD and Stein R were responsible for the research design and conception, data acquisition, analysis and interpretation, manuscript writing and critical revision based on significant intellectual content.

## Figures and Tables

**Table 1:** Baseline characteristics of patients with COVID-19 in different studies.

Study	Country	Patients	Mean age (y)	% Male	% Hypertension	% Chronic cardiac disease	% Cerebrovascular disease
Arentz et al. (68)	United States	Critically ill (N=21)	70	52	NA	43	NA
Bean et al. (31)	United Kingdom	All (N=1,200)	68	57	54	22	NA
Borba et al. (79)	Brazil	All (N=81)	51	75	46	9	NA
Cao et al. (49)	China	Critically ill (N=199)	58 (49-68)*	60	NA	NA	7
Chen et al. (51)	China	All (N=99)	55	68	NA	20	20
Chen et al. (50)	China	Dead (N=38)	70 (65-81)*	71	40	11	8
Docherty et al. (82)	United Kingdom	All ( N=20,133)	73 (58-82)*	60	NA	31	NA
Geleris et al. (69)	United States	All (N=1,376)	NA	57	32	NA	NA
Goldman et al. (81)	Multicenter	All (N=397)	NA	64	50	NA	NA
Goyal et al. (70)	United States	All (N=393)	62	61	50	14	NA
Grasseli et al. (76)	Italy	Critically ill (N=1,591)	63	82	49	21	NA
Grein et al. (80)	Multicenter	Critically ill (N=53)	68 (48-71)*	75	25	NA	NA
Guan et al. (47)	China	All (N=1,099)	47	58	15	3	1
Guo et al. (52)	China	All (N=187)	59	49	33	11	NA
Hou et al. (53)	China	All (N=101)	51	44	21	11	3
Huang et al. (54)	China	All (N=41)	49	73	15	15	NA
Itelman et al. (77)	Israel	All (N=162)	52	65	30	7	NA
Ji et al. (55)	China	All (N=101)	51	48	20	7	7
Li et al. (56)	China	Dead (N=25)	73	40	64	32	16
Li et al. (57)	China	All (N=103)	70 (62-78)*	58	54	25	17
Liang et al. (58)	China	Critically ill (N=131) Noncritically ill (N=1,459)	62 48	NA NA	41 15	10 3	8 1
Mao et al. (59)	China	All (N=214)	53	41	24	7	7
Mercuro et al. (71)	United States	All ( N=90)	60	51	53	21	NA
Mi et al. (60)	China	(All=10)	68	20	4	NA	NA
Myers et al. (72)	United States	All (N =377)	61 (50-73)*	56	44	6	NA
Nahum et al. (78)	France	Critically ill (N=34)	62	78	38	9	NA
Price-Haywood et al. (73)	United States	All (N=3,481)	54	40	31	8	NA
Richardson et al. (74)	United States	All (N=5,700)	63	60	57	18	NA
Shi et al. (61)	China	All (N=416)	64	49	31	15	5
Singh & Khan (75)	United States	All (N=2,530)	52	38	40	9	NA
Sun et al. (62)	China	Discharged (N=123) Dead (N=121)	67 (64-72)* 72 (66-78)*	42 68	50 63	12 17	NA NA
Xie et al. (63)	China	Dead (N=168)	70 (64-78)*	NA	50	23	4
Yang et al. (64)	China	Critically ill (N=52)	59.7	67	NA	10	13.5
Yu et al. (65)	China	All (N=421)	47	53	17	3	1
Wang et al. (46)	China	All (N=138)	56	54	31	15	5
Wu et al. (66)	China	All (N=201)	51 (43-60)*	64	19	4	NA
Zhang et al. (67)	China	All (N=140)	57	51	30	5	2.1

* Median age (interquartile range).

**Table 2:** Summary of results of analysis on use of RAAS inhibitors in hypertensives with COVID-19.

Study	Country	Design	Participants (N)	Men / Women (N)	Comparison	Endpoint	Results
Adrish et al. (12)	United States	Observational	469	279 / 190	ACEi / ARB *vs.* non-ACEi / ARB	Survival time from admission to disposition	ACEi / ABR: ↑15 days (95% CI, 11-17) *vs.* 12 days (95% CI, 11-13); *p*=0.0062.
Bae et al. (15)	South Korea	Observational	610	230 / 280	ACEi / ARB *vs.* other drug	Risk of mortality	No differenceOR 1.00; 95% CI, 0.46 to 2.16).
Bean et al. (32)	United Kindgom	Observational	1,200	686 / 514	ACEi / ARB *vs.* non-ACEi / ARB	Death or transfer to a ICU for organ support within 21-days of symptom onset	ACEi / ABR: ↓Adjusted OR 0.63; 95% CI, 0.47 to 0.84; *p*<0.01†.
Bravi et al. (33)	Italy	Retrospective case-control	1,603	758 / 844	ACEi / ARBs *vs.* non- ACEi / ARBs	Very severe/lethal COVID-19	ACEi or ABR: No differenceOR 0.87; 95% CI, 0.50 to 1.49; *p*=0.6.ACEi: No differenceOR 0.82; 95% CI, 0.49 to 1.36; *p*=0.4.ARB: No differenceOR 0.83; 95% CI, 0.50 to 1.40; *p*=0.5.
Cannata et al. (34)	Italy	Observational	397	NA	ACEi/ARB discontinuation *vs.* no therapyACEi/ARB continuation *vs.* discontinuationACEi/ARB continuation *vs.* no therapyACEi/ARB continuation *vs.* discontinuation / no therapy	All-cause mortality	ACEi/ARB discontinuation *vs.* no therapy: No differenceAdjusted OR 1.11; 95% CI, 0.36 to 3.45.ACEi/ARB continuation *vs.* discontinuation: No differenceAdjusted OR 0.17; 95% CI, 0.03 to 1.00.ACEi/ARB continuation *vs.* no therapy: ↓Adjusted OR 0.05; 95% CI, 0.01 to 0.54.ACEi/ARB continuation *vs.* discontinuation / no therapy: ↓Adjusted OR 0.14; 95% CI, 0.03 to 0.66.
Conversano et al. (7)	Italy	Observational	191	131 / 60	ACEi / ARB *vs.* non-ACEi / ARB	All-cause mortality	No differenceHR 0.50 (95% CI, 0.20 to 1.20; *p*=0.13).
Covino et al. (8)	Italy	Observational	166	109 / 57	ACEi / ARB *vs.* non-ACEi / ARB	Death, and combined of death/admission to ICU	No difference**Death:** 18% *vs.* 16.3%; *p*=0.79.**Combined of death/admission to ICU:** 34.2% *vs.* 23.6%; *p*=0.16.
de Abajo et al. (35)	Spain	Observational	12,529	7,645 / 4,884	ACEi / ARB *vs.* other antihypertensive drugs	COVID-19 requiring admission to hospital	ACEi: No differenceAdjusted OR 0.80; 95% CI, 0.64 to 1.00.ARB: No differenceAdjusted OR 1.10; 95% CI, 0.88 to 1.37.
De Spiegeleer et al. (36)	Belgium	Observational	154	51 / 103	ACEi / ARBs *vs.* non- ACEi / ARBs	Asymptomatic status, and serious clinical outcome	No difference**Asymptomatic status:**OR 2.72; 95% CI, 0.59 to 25.1; *p*=0.24.**Serious clinical outcome:** OR 0.48; 95% CI, 0.10 to 1.97; *p*=0.31.
Felice et al. (37)	Italy	Observational	133	86 / 47	ACEi / ARB *vs.* other antihypertensive drugs	Hospital admission, oxygen therapy, admission to ICU/sICU, non-invasive ventilation, and death	No difference**Hospital admission:** adjusted OR 0.39; 95% CI, 0,05 to 2.94; *p*=0.36.**Oxygen therapy:** adjusted OR 0.51; 95% CI, 0.15 to 1.78; *p*=0.29.**Non-invasive ventilation:** adjusted OR 0.58; 95% CI, 0.21 to 1.60; *p*=0.29.**Death:** adjusted OR 0.56; 95% CI, 0.17 to 1.83; *p*=0.34.ACEi / ARB: ↓ **Admission to ICU/sICU**Adjusted OR 0.25; 95% CI, 0.09 to 0.66; *p*=0.006.
Fosbøl et al. (27)	Denmark	Observational	4,480		ACEi / ARB *vs.* non-ACEi / ARB	Composite outcome of death or severe COVID-19	No differenceAdjusted HR 1.04; 95% CI, 0.89 to 1.23.
Gao et al. (38)	China	Observational	850	443 / 407	ACEi / ARB *vs.* other antihypertensive drugs	Mortality rates	No differenceAdjusted HR 0.85; 95% CI, 0.28 to 2.58; *p*=0.77.
Hakeam et al. (20)	Saudi Arabia	Observational	338	201 / 137	ACEi / ARB *vs.* non-ACEi / ARB	ICU admission, ICU admission within 24 hours of hospitalization, ICU stay (days), and ICU death	No difference**ICU admission:** 28.2% *vs.* 35.5%; *p*=0.19.**ICU admission within 24 hours of hospitalization:** 63.8% *vs.* 51.5%; *p*=0.23.**ICU stay (days):** 10.5 *vs.* 7; *p*=0.13.**ICU death:** 21.7% *vs.* 21.2%; *p*=0.58.
Hippisley-Cox et al. (39)	England	Observational	19,486	9,376 / 10,110	ACEi / ARBs *vs.* non- ACEi / ARBs	Risk of COVID-19, risk of ICU care	ACEi/ARBs**Risk of COVID-19:** ↓Adjusted HR 0.71; 95% CI, 0.67 to 0.74.**Risk of ICU care:** No differenceAdjusted HR 0.89; 95% CI, 0.75 to 1.06.
Huang et al. (6)	China	Observational	50	27 / 23	RAAS inhibitors *vs.* non-RAAS inhibitors	In hospital mortality	No difference0% *vs.* 6.67%; *p*=0.51
Jung et al. (17)	Korea	Observational	5,179	2,295 / 2,884	ACEi / ARB *vs.* non-ACEi / ARB	Risk of mortality	No differenceAdjusted OR 0.88; 95% CI, 0.53 to 1.44; *p*=0.60).
Khan et al. (24)	Scotland	Observational	88	50 / 38	ACEi / ARB *vs.* non-ACEi / ARB	Critical care admission, intubated and ventilated, and in-patient mortality	No difference**Critical care admission:** 33.3% *vs.* 14.7%; *p*=0.08.**Intubated and ventilated:** 18.5% *vs.* 11.5%; *p*=0.58.**In-patient mortality:** 18.5% *vs.* 22.9%; *p*=0.85.
Kim et al. (18)	Korea	Observational	1,378,052	649,153 / 728,899	ARB *vs.* non-ARB	Risk of COVID-19	ARB: ↓Adjusted RR 0.75; 95% CI, 0.59 to 0.96).
Kocayigit et al. (25)	Turkey	Observational	169	79 / 90	ACEi / ARB *vs.* other antihypertensive drugs	In-hospital mortality	No differenceOR 0.53; 95% CI, 0.13 to 2.14; *p*=0.37.
Lam et al. (13)	United States	Observational	614	338 / 276	ACEi/ARB continuation in the hospital *vs.* discontinuation	Mortality, and ICU admission	ACEi/ARB continuation: ↓**Mortality:** OR 0.21; 95% CI, 0.10 to 0.45; adjusted *p*=0.001.**ICU admission:** OR 0.35; 95% CI, 0.19 to 0.64; *p*=0.001.
Li et al. (2)	China	Observational	362	173 / 189	ACEi / ARB *vs.* non-ACEi / ARB	Severe and non-severe infections, non-survivors and survivors	No difference**Severe and non-severe infections:** 32.9% *vs.* 30.7%; *p*=0.65.**Non-survivors and survivors:** 27.3% *vs.* 33.0%; *p*=0.34.
Liu et al. (3)	China	Observational	157	73 / 84	ACEi / ARBs *vs.* CCBs	Chest computed tomography time, and hospitalization time	No difference**Chest computed tomography time:**HR 0.73; 95% CI, 0.45 to 1.2; *p*=0.87.**Hospitalization time:** HR 1.06; 95% CI, 0.44 to 1.1, *p*=0.83.
Lopes et al. (28)	Brazil	Randomized clinical trial	659	389 / 270	ACEi/ARB continuation in the hospital *vs.* discontinuation	Primary outcome: number of days alive and out of the hospital; secondary outcome: all-cause death	**Primary outcome:** No difference22.9 *vs.* 21.9; *p*=0.09.**Secondary outcome:** No difference2.8% *vs.* 2.7%; *p*=0.95.
Mancia et al. (10)	Italy	Observational	6,272	3,969 / 2,303	ACEi / ARBs *vs.* non- ACEi / ARBs	Association with COVID-19, and severe or fatal course of the disease	ARB: No difference**Association with COVID-19:** adjusted OR 0.95; 95% CI, 0.86 to 1.05.**Severe or fatal course of the disease:** adjusted OR 0.83; 95% CI, 0.63 to 1.10.ACEi: No difference**Association with COVID-19:** adjusted OR 0.96; 95% CI, 0.87 to 1.07.**Severe or fatal course of the disease:** adjusted OR 0.91; 95% CI, 0.69 to 1.21.
Mehta et al. (40)	United States	Observational	3,470	1,718 / 1,752	ACEi / ARB *vs.* non-ACEi / ARB	Admitted to hospital, admitted to ICU, and use of ventilator	ACEi*:*↑ **Admitted to hospital:** OR 1.84; 95% CI, 1.22 to 2.79.↑ **Admitted to ICU:** OR 1.77; 95% CI, 1.07 to 2.92.**Use of ventilator:** OR 1.35; 95% CI, 0.74 to 2.47.ARB*:*↑ **Admitted to hospital:** OR 1.61; 95% CI, 1.04 to 2.50.**Admitted to ICU:** OR 1.16; 95% CI, 0.67 to 2.02.**Use of ventilator:** OR 1.12; 95% CI, 0.59 to 2.12.
Pan et al. (41)	China	Observational	282	143 / 139	ACEi / ARB *vs.* other antihypertensive drugs	All-cause mortality, and proportion of critically ill	ACEi / ABR: ↓ **All-cause mortality**9.8% *vs.* 26.1%; *p*=0.03**Proportion of critically ill:** No difference31.7% *vs.* 43.2%; *p*=0.16.
Reynolds et al. (14)	United States	Observational	4,357	2,214 / 2,143	ACEi / ARB *vs.* non-ACEi / ARB	Severe COVID-19*	ACEi: No differenceMedian difference -3.3; 95% CI, -8.2 to 1.7.ARB: No differenceMedian difference 0.1; 95% CI, -4.8 to 4.9.
Sardu et al. (11)	Italy	Observational	62	41 / 21	ACEi / ARBs *vs.* CCBs	Hospital admission at ICU, mechanical ventilation, cardiac injury, and death	No difference
Savarese et al. (21)	Sweden	Observational	1,387,746	722,900 / 664,846	ACEi / ARB *vs.* non-ACEi / ARB	Risk of hospitalization/death for Covid-19 in the overall population, and risk of all-cause death in patients with COVID-19	ACEi/ARB: ↓**Risk of hospitalization/death for COVID-19 in the overall population:** Adjusted OR 0.86; 95% CI, 0.82 to 0.91.**Risk of all-cause death in patients with COVID-19:** Adjusted HR 0.90; 95% CI, 0.83 to 0.97).
Şenkal et al. (26)	Turkey	Observational	611	363 / 248	ACEi / ARB *vs.* other antihypertensive drugs	Severe disease	ACEi*:* ↓OR 0.37, 95% CI, 0.15 to 0.87; *p*=0.03.ARB: No differenceOR 0.60, 95% CI, 0.27 to 1.36; *p*=0.31.
Seo et al. (16)	South Korea	Observational	4,932	2,142 / 2,790	ACEi / ARB *vs.* non-ACEi / ARB	COVID-19 infection, and death	No difference**COVID-19 infection:** Adjusted OR 0.98; 95% CI, 0.85 to 1.13.**Death:** Adjusted OR 0.87; 95% CI, 0.55 to 1.40.
Son et al. (19)	South Korea	Observational	2,847	1,449 / 1.398	ACEi / ARB *vs.* non-ACEi / ARB	COVID-19 infection, long-term hospitalization, ICU admission, high-flow oxygen therapy, and death	No difference**COVID-19 infection:** Adjusted OR 1.16; 95% CI, 0.96 to 1.41.**Long-term hospitalization:** Adjusted OR 0.86; 95% CI, 0.53 to 1.40.**ICU admission:** Adjusted OR 1.51; 95% CI, 0.40 to 5.70; *p*>0.05.**High-flow oxygen therapy:** Adjusted OR 0.66; 95% CI, 0.28 to 1.62; *p*>0.05.**Death:** Adjusted OR 1.36; 95% CI, 0.51 to 3.66; *p*>0.05.
Tetlow et al. (23)	London	Observational	557	320 / 237	ACEi / ARB *vs.* non-ACEi / ARB	Macrovascular thrombus, acute kidney injury, and in-hospital mortality	No difference**Macrovascular thrombus:** Overlap propensity score - RR 1.05; 95% CI, 0.48 to 2.31.**Acute kidney injury:** Overlap propensity score - RR 1.04; 95% CI, 0.71 to 1.52.**In-hospital mortality:** Overlap propensity score - RR 1.04; 95% CI, 0.80 to 1.36.
COVID-19 RISk and Treatments Collaboration (9)	Italy	Observational	4,069	1,560 / 2,509	ACEi / ARB *vs.* non-ACEi / ARB	In-hospital death	ACEi: No differenceAdjusted HR 0.96; 95% CI, 0.77 to 1.20.ARB: No differenceAdjusted HR 0.89; 95% CI, 0.67 to 1.19.
Vila-Corcoles et al. (22)	Spain	Observational	34,936	16,805 / 18,131	ACEi / ARB *vs.* non-ACEi / ARB	Risk of COVID-19	ACEi: No differenceHR 0.83; 95% CI, 0.61 to 1.13/ *p*=0.24.ARB: No differenceHR 0.67; 95% CI, 0.44 to 1.01; *p*=0.05.
Wang et al. (4)	China	Observational	210	100 / 110	ACEi / ARB *vs.* non-ACEi / ARB	Death during hospitalization, days of hospital stay, adverse events	No difference**Death during hospitalization:** 8.64% *vs.* 3.88%; *p*=0.15.**Days of hospital stay:** 17.00 *vs.* 17.00; *p*=0.82.**Adverse events:** 5.24% *vs.* 6.17%; *p*=0.63.
Xu et al. (42)	China	Observational	101	53 / 48	ACEi / ARB *vs.* other antihypertensive drugs	In-hospital mortality, ICU admission, or invasive mechanical ventilation	No difference**In-hospital mortality:** 28% *vs.* 34%; *p*=0.46.**ICU admission:** 20% *vs.* 28%; *p*=0.37.**Invasive mechanical ventilation:** 18% *vs.* 26%; *p*=0.31.
Yang et al. (5)	China	Observational	126	62 / 64	ACEi / ARB *vs.* non-ACEi / ARB	Proportion of critical patients, and death rate	No difference**Proportion of critical patients:** 9.3% *vs.* 22.9%; *p*=0.06.**Death rate:** 4.7% *vs.* 13.3%; *p*=0.21.
Zhang et al. (43)	China	Observational	1,128	603 / 525	ACEi / ARB *vs.* non-ACEi / ARB	Risk for all-cause mortality, and COVID-19 mortality	ACE / ARB: ↓**Risk for all-cause mortality: **Adjusted HR 0.42; 95% CI, 0.19 to 0.92; *p*=0.03.**COVID-19 mortality:** Adjusted HR 0.37; 95% CI, 0.15 to 0.89; *p*=0.03.
Zhou et al. (44)	China	Observational	3,572	NA	ACEi / ARB *vs.* other antihypertensive drugs	28-day all-cause death of COVID-19	ACEi / ARB: ↓Adjusted HR 0.39; 95% CI, 0.26 to 0.58; *p*<0.001.

ACEi, angiotensin-converting enzyme inhibitor; ARB, angiotensin II receptor blocker; CCB, calcium-channel blocker; THZ, thiazide diuretic; ICU, intensive care unit; sICU, semi-intensive care unit; HR, hazard ratio; OR, odds ratio; RR, risk ratio.

* Admission to the ICU, requirement of noninvasive mechanical ventilation, or death.

† After adjustment for age, sex, and comorbidities
